# Author Correction: Salivary gland organoid culture maintains distinct glandular properties of murine and human major salivary glands

**DOI:** 10.1038/s41467-024-51830-8

**Published:** 2024-09-13

**Authors:** Yeo-Jun Yoon, Donghyun Kim, Kwon Yong Tak, Seungyeon Hwang, Jisun Kim, Nam Suk Sim, Jae-Min Cho, Dojin Choi, Youngmi Ji, Junho K. Hur, Hyunki Kim, Jong-Eun Park, Jae-Yol Lim

**Affiliations:** 1 Department of Otorhinolaryngology, Yonsei University College of Medicine, Seoul, South Korea; 2 Graduate School of Medical Science and Engineering, Korean Advanced Institute of Science and Technology, Daejeon, South Korea; 3 National Institute of Dental and Craniofacial Research, NIH, Bethesda, MD USA; 4 Department of Genetics, College of Medicine, Graduate School of Biomedical Science & Engineering, Hanyang University, Seoul, South Korea; 5 Department of Pathology, Yonsei University College of Medicine, Seoul, South Korea

Correction to: *Nature Communications* 10.1038/s41467-022-30934-z, published online 07 June 2022

In this article the author name Youngmi Ji was incorrectly written as Yongmi Ji. The original article has been corrected.

