# Supplementary material for: Salivary gland organoid culture maintains distinct glandular properties of murine and human major salivary glands
Source: Nat Commun. 2022 Jun 7;13:3291. doi: 10.1038/s41467-022-30934-z (PMC9174290; doi:10.1038/s41467-022-30934-z)
Supplement: Supplementary file 1 — Supplementary Information [file 41467_2022_30934_MOESM1_ESM.pdf]

Supplementary Information

Salivary gland organoid culture maintains distinct glandular properties of murine and human major salivary glands

Yeo-Jun Yoon[1†], Donghyun Kim[1†], Kwon Yong Tak[2], Seungyeon Hwang[1], Jisun Kim[1], Nam Suk Sim[1], Jae-Min Cho[1], Dojin Choi[1], Yongmi Ji[3], Junho K. Hur[4], Hyunki Kim[5], Jong-Eun Park[2], and Jae-Yol Lim[1*]

[1]Department of Otorhinolaryngology, Yonsei University College of Medicine, Seoul, South Korea

[2]Graduate School of Medical Science and Engineering, Korean Advanced Institute of Science and Technology, Daejeon, South Korea

[3]National Institute of Dental and Craniofacial Research, NIH, Bethesda, MD, USA

[4]Department of Genetics, College of Medicine, Graduate School of Biomedical Science & Engineering, Hanyang University, Seoul, South Korea

[5]Department of Pathology, Yonsei University College of Medicine, Seoul, South Korea

[†]These authors contributed equally.

[*]Correspondence: jylimmd@yuhs.ac

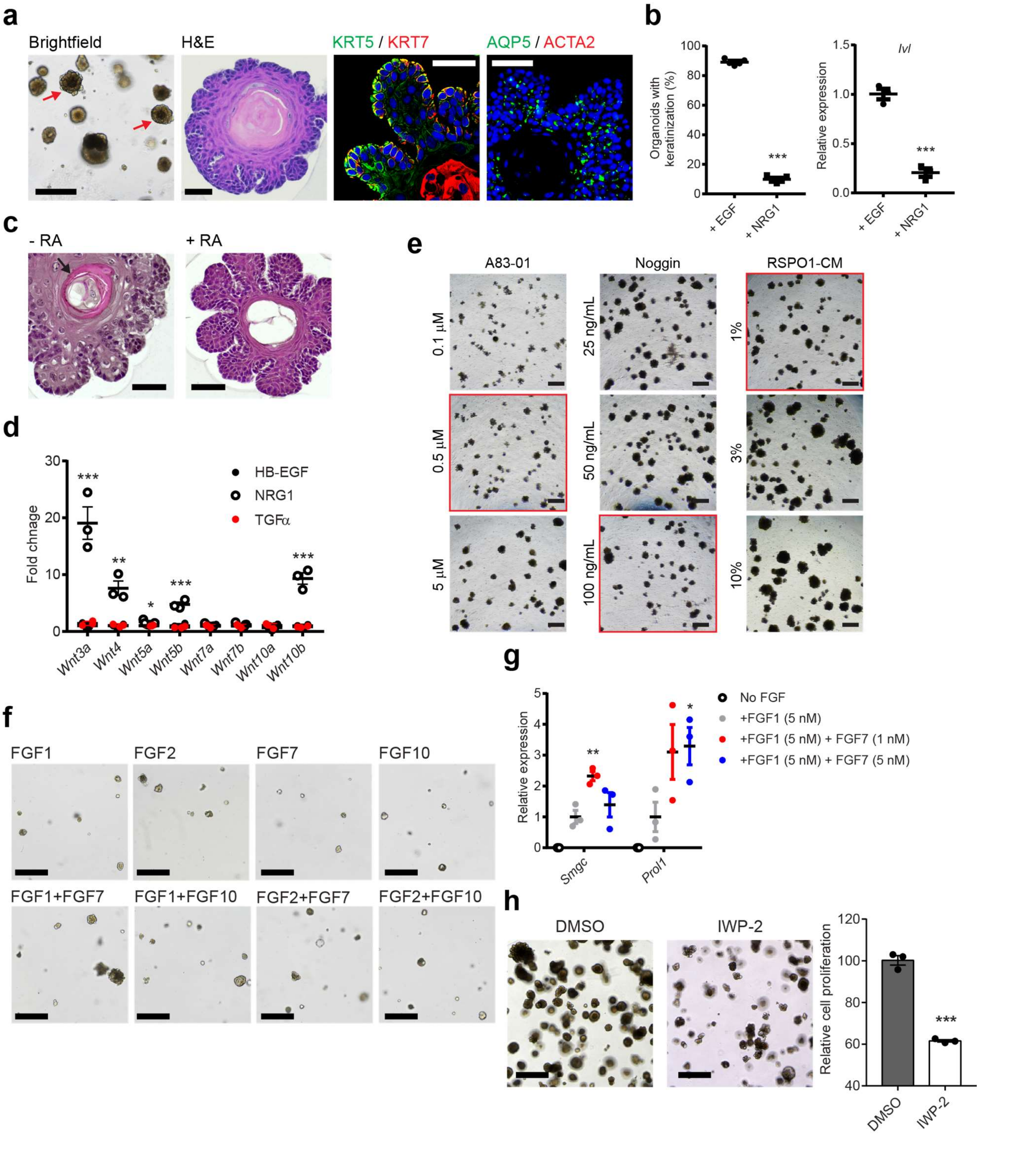

Supplementary Figure 1. Optimization of murine salivary gland GEM for sustained organoid culture.

(a) mSMG organoids were cultured in EGF-containing media and subjected to brightfield, H&E, or IF imaging for ductal (KRT5/KRT7) or acinar/myoepithelial (AQP5/ACTA2) markers. Red arrows indicate organoids with keratinized core. Scale bars indicate 500 μm for brightfield images and 50 μm for H&E and IF images. **(b)** Proportions of keratinized organoids (left) and expressions of Involucrin mRNA (right) were compared between the organoids cultured in EGF- or NRG1-containing media (n = 3). **(c)** H&E staining of mSMG organoids cultured in the GEM with or without retinoic acid (RA). Black arrow indicates the keratin core. Scale bar indicates 50 μm. **(d)** The expression of WNT gene family members was assessed in the organoids cultured in media containing EGF, HB-EGF, NRG1,

or TGFα. The gene expression of organoids cultured in EGF-containing media was normalized as 1 (n = 3). **(e)** Dose-dependent effect of A83-01 (left), Noggin (middle), and RSPO1-CM (right) on organoid growth was determined with brightfield images. Scale bar indicates 200 μm. The selected conditions used in mouse GEM are highlighted with red outlines. **(f)** FGF-induced growth support was observed using brightfield microscopy. FGF1, FGF2, FGF7, or FGF10 were added solely or in combination to NRG1-based media. Scale bar indicates 500 μm. **(g)** Expression of the acinar-related gene (*Smgc* and *Prol1*) was measured at different compositions of FGF1 or FGF7. Expressions were not detected in the organoids cultured without FGFs (n = 3). **(h)** mSMG organoid were cultured in GEM supplemented with DMSO or 2 μM IWP-2 for 7 days. Organoid growth was determined with brightfield images (left) and quantified via luminescence (right, n = 3). Scale bar indicates 200 μm. Data are representative of at least three independent experiments, and presented as mean ± SEM, * $p < 0.05$, ** $p < 0.01$, *** $p < 0.001$. Source data are provided as a Source Data file.

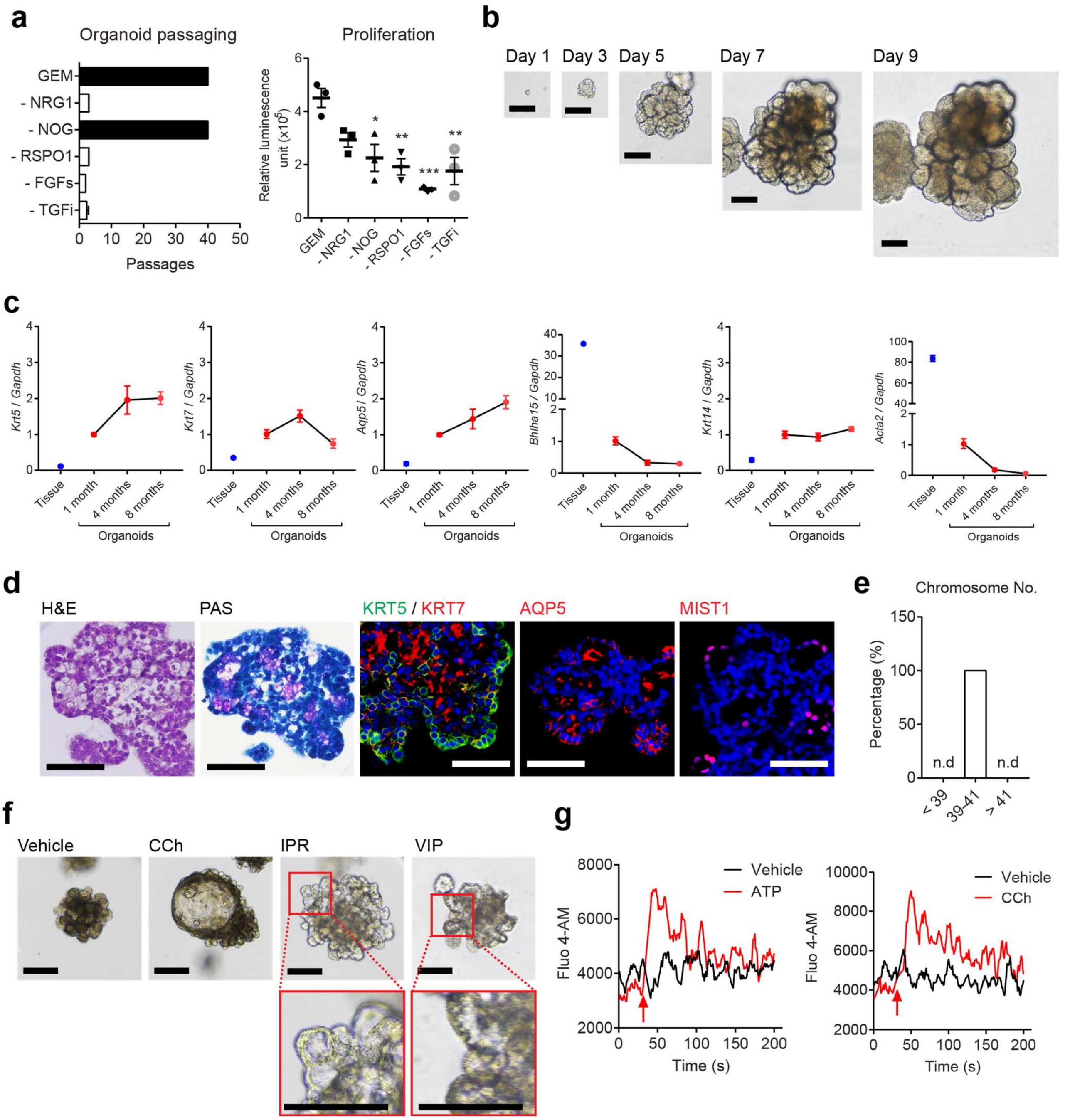

Supplementary Figure 2. Gene expression and functionality of long-term cultured murine salivary gland organoids.

(a) mSMG organoids were maintained in the GEM or GEM lacking each factor and their maximum passage number (left) and proliferation capacity (right, n = 3) were evaluated. **(b)** mSMG organoids were maintained for longer period (passage 30). The growth of a single organoid was tracked in time-lapse of images obtained at each time point. **(c)** mRNA expression of ductal (*Krt5*, *Krt7*), acinar (*Aqp5*, *Bhlha15*), and myoepithelial (*Krt14, Acta2*) was determined in mouse SMG tissues and organoids that were cultured for different periods. Expressions in organoids cultured for 1 month were normalized as 1 (n = 3). **(d-g)** mSMG organoids maintained for 30 passages were used for assessment of marker expression, genetic stability, and functionality. **(d)** H&E, PAS, and IF staining indicated an intact ductal structure (KRT5, KRT7) and the presence of acinar cells (AQP5, MIST1). **(e)** These organoids exhibited no chromosomal

abnormalities, as determined by karyotyping. **(f)** The functionality of organoids was observed via neurotransmitter-mediated organoid swelling. DMSO was used as a negative control (vehicle). Red boxes indicate swollen sites in organoids treated with IPR and VIP. **(g)** ATP (left)- or CCh (right)-mediated increases in calcium influx were assessed via Fluo 4-AM. Data are representative of three independent experiments, and presented as mean ± SEM. * $p < 0.05$, ** $p < 0.01$, *** $p < 0.001$, n.d not detected. Scale bars indicate 100 μm in **(b)**, 50 μm in **(d)**, and 200 μm in **(f)**. Source data are provided as a Source Data file.

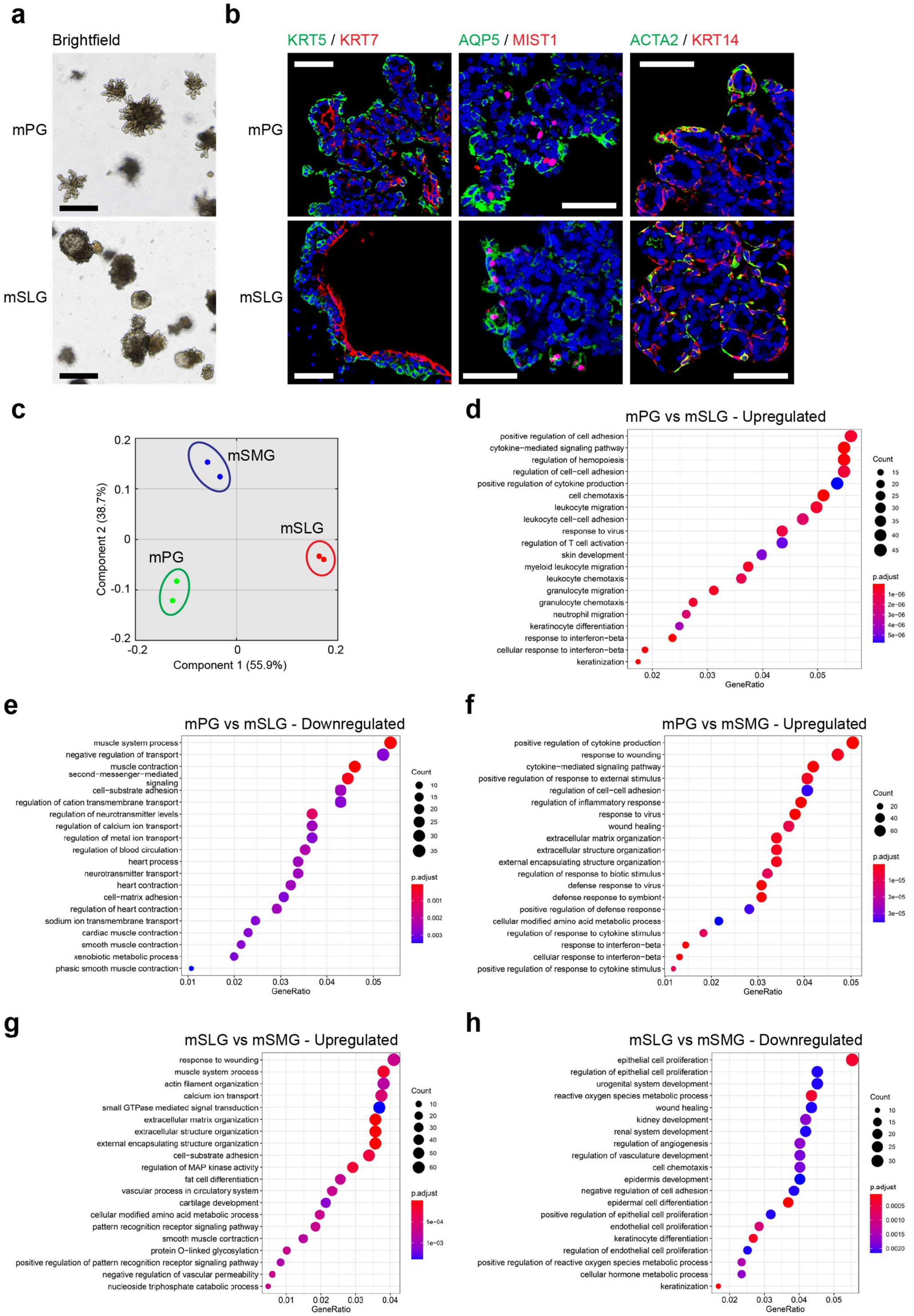

a
Brightfield
mPG
mSLG
b
KRT5 / KRT7
AQP5 / MIST1
ACTA2 / KRT14
mPG
mSLG
c
mSMG
mSLG
mPG
Component 1 (55.9%)
Component 2 (38.7%)
d
mPG vs mSLG - Upregulated
positive regulation of cell adhesion
cytokine−mediated signaling pathway
regulation of hemopoiesis
regulation of cell−cell adhesion
positive regulation of cytokine production
cell chemotaxis
leukocyte migration
leukocyte cell−cell adhesion
response to virus
regulation of T cell activation
skin development
myeloid leukocyte migration
leukocyte chemotaxis
granulocyte migration
granulocyte chemotaxis
neutrophil migration
keratinocyte differentiation
response to interferon−beta
cellular response to interferon−beta
keratinization
GeneRatio
Count
p.adjust
e
mPG vs mSLG - Downregulated
muscle system process
negative regulation of transport
muscle contraction
second−messenger−mediated signaling
cell-substrate adhesion
regulation of cation transmembrane transport
regulation of neurotransmitter levels
regulation of calcium ion transport
regulation of metal ion transport
regulation of blood circulation
heart process
neurotransmitter transport
heart contraction
cell-matrix adhesion
regulation of heart contraction
sodium ion transmembrane transport
cardiac muscle contraction
smooth muscle contraction
xenobiotic metabolic process
phasic smooth muscle contraction
f
mPG vs mSMG - Upregulated
positive regulation of cytokine production
response to wounding
cytokine−mediated signaling pathway
positive regulation of response to external stimulus
regulation of cell−cell adhesion
regulation of inflammatory response
response to virus
wound healing
extracellular matrix organization
extracellular structure organization
external encapsulating structure organization
regulation of response to biotic stimulus
defense response to virus
defense response to symbiont
positive regulation of defense response
cellular modified amino acid metabolic process
regulation of response to cytokine stimulus
response to interferon−beta
cellular response to interferon−beta
positive regulation of response to cytokine stimulus
g
mSLG vs mSMG - Upregulated
response to wounding
muscle system process
actin filament organization
calcium ion transport
small GTPase mediated signal transduction
extracellular matrix organization
extracellular structure organization
external encapsulating structure organization
cell-substrate adhesion
regulation of MAP kinase activity
fat cell differentiation
vascular process in circulatory system
cartilage development
cellular modified amino acid metabolic process
pattern recognition receptor signaling pathway
smooth muscle contraction
protein O−linked glycosylation
positive regulation of pattern recognition receptor signaling pathway
negative regulation of vascular permeability
nucleoside triphosphate catabolic process
h
mSLG vs mSMG - Downregulated
epithelial cell proliferation
regulation of epithelial cell proliferation
urogenital system development
reactive oxygen species metabolic process
wound healing
kidney development
renal system development
regulation of angiogenesis
regulation of vasculature development
cell chemotaxis
epidermis development
negative regulation of cell adhesion
epidermal cell differentiation
positive regulation of epithelial cell proliferation
endothelial cell proliferation
keratinocyte differentiation
regulation of endothelial cell proliferation
positive regulation of reactive oxygen species metabolic process
cellular hormone metabolic process
keratinization

Supplementary Figure 3. Characteristics of murine PG and SLG organoids.

(a-b) Murine PG (top) or SLG (bottom) organoids were maintained in the GEM and differentiated in the DAM for 3 days. Then, the organoids were harvested and assessed for morphology using brightfield microscopy **(a)** or via IF staining for the specific expression of ductal (KRT5, KRT7), acinar (AQP5, MIST1), and myoepithelial (ACTA2, KRT14) markers **(b)**. Scale bars indicate 500 μm in **(a)** and 50 μm in **(b)**. **(c)** Multidimensional scaling plot showing the separation of mSMG, mPG, and mSLG organoids. **(d-e)** Gene ontology enrichment analysis of genes upregulated **(d)** or downregulated **(e)** when mPG organoids were compared versus mSLG organoids. **(f)** Gene ontology enrichment analysis of genes upregulated when mPG organoids were compared versus mSMG organoids. We observed no downregulated pathways with statistical significance. **(g-h)** Gene ontology enrichment analysis of genes upregulated **(g)** or downregulated **(h)** when mSLG organoids were compared versus mSMG organoids. The microscopic data are representative of three independent experiments.

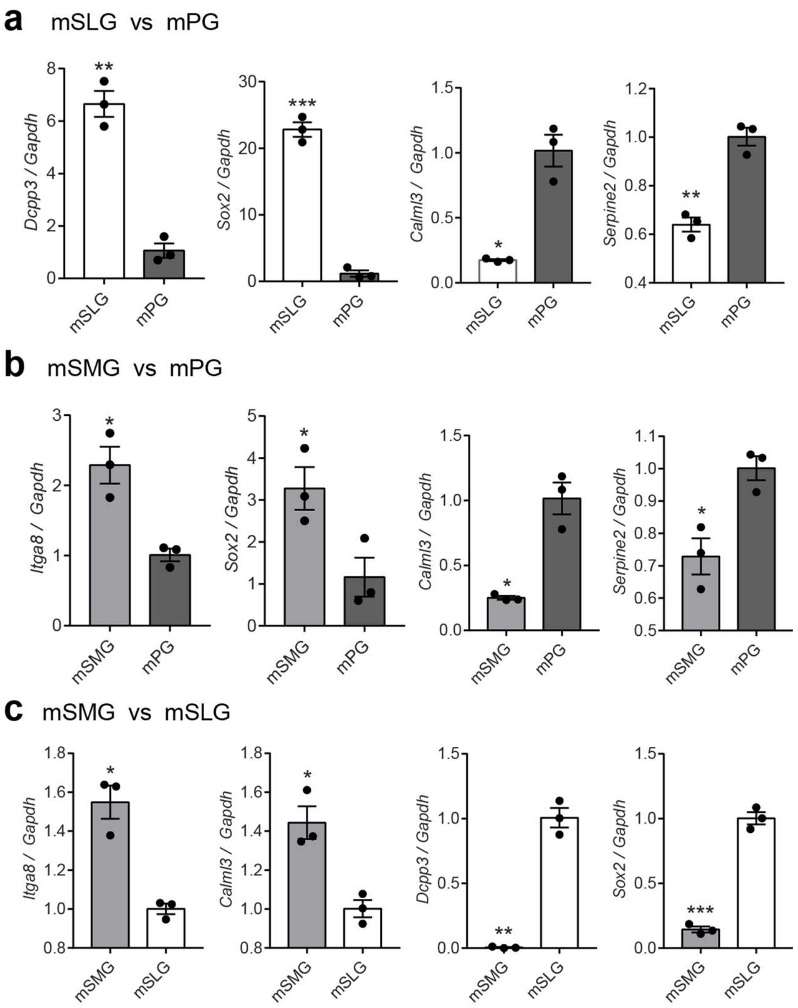

Supplementary Figure 4. qRT-PCR of DEGs from murine PG, SMG, and SLG organoids.

(a) The expressions of *Dcpp3*, *Sox2*, *Calml3*, and *Serpine2*, DEGs between mSLG organoids and mPG organoids, were validated with qRT-PCR. **(b)** The expressions of *Itga8*, *Sox2*, *Calml3*, and *Serpine2*, DEGs between mSMG organoids and mPG organoids, were validated with qRT-PCR. **(c)** The expressions of *Itga8*, *Calml3*, *Dcpp3*, and *Sox2*, DEGs between mSMG organoids and mSLG organoids, were validated with qRT-PCR. Data are representative of three independent experiments, and presented as mean ± SEM, * $p < 0.05$, ** $p < 0.01$, *** $p < 0.001$. Source data are provided as a Source Data file.

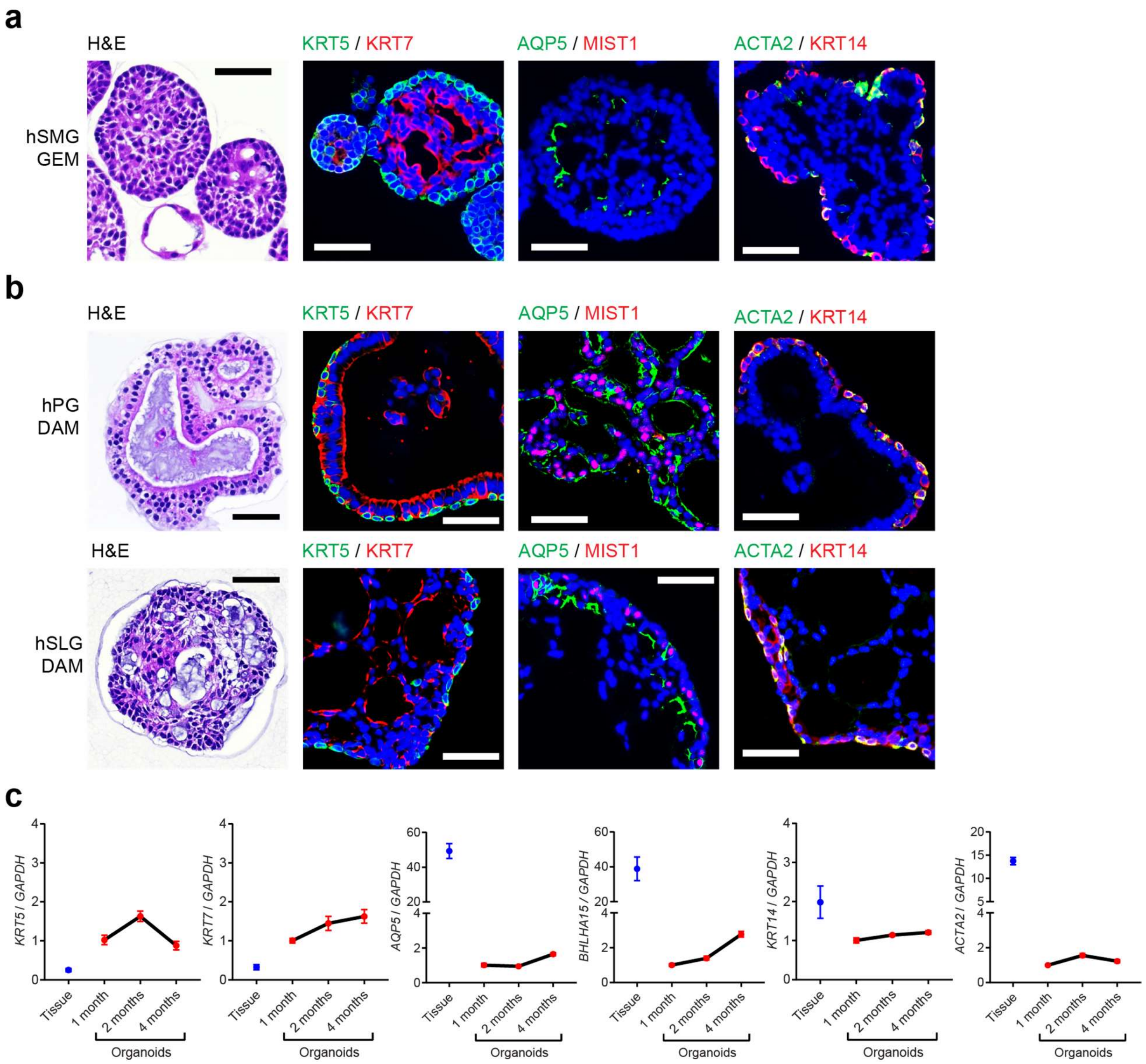

Supplementary Figure 5. Gene expression of organoids from three major human salivary glands.

(a) Human SMG organoids were maintained in the GEM. Harvested organoids were subjected to H&E or IF staining for duct (KRT5/KRT7), acinar (AQP5/MIST1), and myoepithelial (ACTA2/KRT14) markers. Nuclei were stained with Hoechst 33342 (blue) in IF. Scale bars indicate 50 μm. **(b)** Human PG (top) or SLG (bottom) organoids were maintained in the GEM and further incubated in the DAM for 3 days. Harvested organoids were subjected to H&E or IF staining for ductal, acinar, and myoepithelial markers. Nuclei were stained with Hoechst 33342 (blue) in IF. Scale bars indicate 50 μm. **(c)** mRNA expressions of ductal (*KRT5* and *KRT7*), acinar (*AQP5* and *BHLHA15*), and myoepithelial (*KRT14* and *ACTA2*) markers were determined with human SMG tissues and organoids cultured for different periods ($n = 4$). Expressions in organoids cultured for 1 month were normalized as 1. Data represent at least three independent experiments, and are presented as mean ± SEM. Source data are provided as a Source Data file.

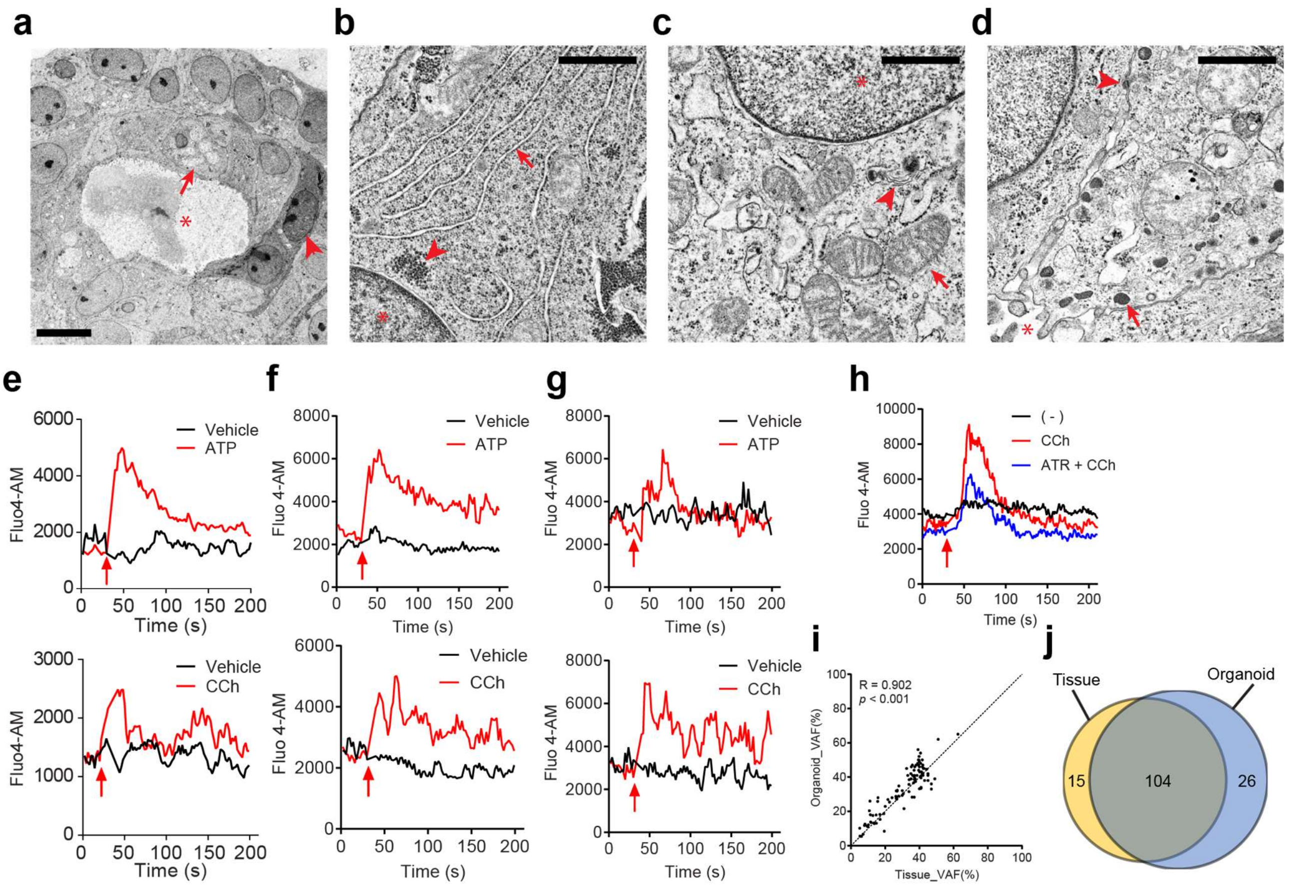

Supplementary Figure 6. Ultrastructure, functionality, and genetic stability of human salivary gland organoids.

(a) TEM images of human SMG organoids with several cell types, including secretory epithelial and myoepithelial cells. Red asterisk indicates internal lumen. Red arrow indicates secretory cells with visible vesicles. Red arrowhead indicates myoepithelial cell. Scale bars indicate 10 μm. **(b-d)** TEM images showing the ultrastructure of organoids. **(b)** Red asterisk indicates nucleus. Red arrow indicates ER, while red arrowheads indicate glycogen deposits observed in myoepithelial cells. **(c)** Red asterisk indicates nucleus. Red arrow indicates a mitochondrion, while a red arrowhead indicates the Golgi apparatus. **(d)** Red asterisk indicates internal lumen. Red arrow indicates a lysosome, while red arrowhead indicates a tight junction. Scale bars indicate 1 μm. **(e-g)** Single-cell suspensions were prepared from differentiated human SMG **(e)**, PG **(f)**, or SLG **(g)** organoids after 1 month of culture in the GEM and another 3 days of culture in the DAM. The calcium influx was assessed using Fluo 4-AM in stimulation with either ATP (top) or CCh (bottom). **(h)** Human SMG organoids maintained for 3 months in the GEM, followed by 3 days in the DAM, were untreated or pretreated with 0.1 μM atropine and stimulated with CCh (10 μM). The calcium influx was assessed using Fluo 4-AM. Red arrows indicate time points at which ATP or CCh were added. **(i)** Comparison of somatic conserved mutations between human SMG tissues and SMG organoids cultured for 3 months using whole exome sequencing. The graph is displayed with corresponding linear regression statistics, including Pearson correlation coefficient (*R*) and p-value. **(j)** Somatic mutations in human SMG tissues (yellow circle) and SMG organoids (blue circle) indicate the conservation of the majority of somatic mutations (n = 104, grey region) between tissues and organoids. Data represent at least three independent experiments. Source data are provided as a Source Data file.

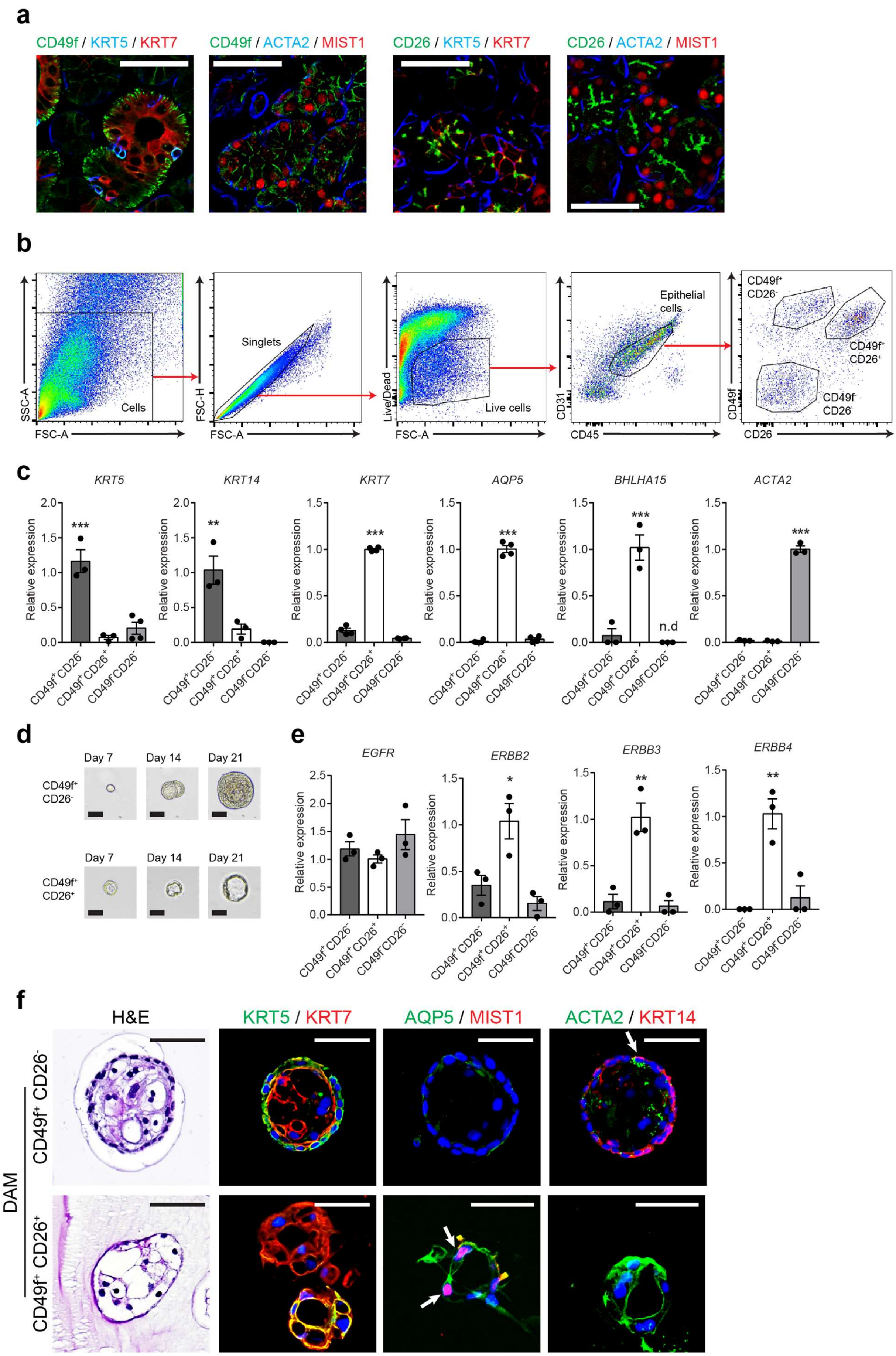

a
CD49f / KRT5 / KRT7
CD49f / ACTA2 / MIST1
CD26 / KRT5 / KRT7
CD26 / ACTA2 / MIST1
b
Cells
SSC-A
FSC-A
Singlets
FSC-H
Live/Dead
Live cells
Epithelial cells
CD31
CD45
CD49f+ CD26-
CD49f+ CD26+
CD49f- CD26-
CD49f
CD26
c
KRT5
KRT14
KRT7
AQP5
BHLHA15
ACTA2
Relative expression
n.d
CD49f+CD26-
CD49f+CD26+
CD49f-CD26-
d
Day 7
Day 14
Day 21
CD49f+ CD26-
CD49f+ CD26+
e
EGFR
ERBB2
ERBB3
ERBB4
Relative expression
f
H&E
KRT5 / KRT7
AQP5 / MIST1
ACTA2 / KRT14
DAM
CD49f+ CD26-
CD49f+ CD26+

Supplementary Figure 7. The expression of CD49f and CD26 markers determines basal or luminal cells in salivary glands.

(a) The tissue expression of CD49f and CD26 was assessed in combination with KRT5/KRT7 and ACTA2/MIST1 via IF staining. Scale bars indicate 50 μm. **(b)** Gating strategy of flow cytometry for the isolation of salivary gland epithelial cell subpopulations. Live singlets were gated out, and the endothelial and immune cells expressing CD31 and CD45, respectively, were excluded. Remaining epithelial cells were further divided into subpopulations based on the expression of the CD49f and CD26 markers. **(c)** $CD49f^{+}CD26^{-}$, $CD49f^{+}$ $CD26^{+}$, and $CD49f^{-}$ $CD26^{-}$ cells from human SMG were isolated via FACS-sorting and assessed for the expression of structural gene markers (n = 3). **(d)** Single-cell suspensions from $CD49f^{+}$ $CD26^{-}$ and $CD49f^{+}$ $CD26^{+}$ populations were subjected to organoid culture. The growth of a single organoid was tracked for 3 weeks. Scale bars indicate 50 μm. **(e)** $CD49f^{+}$ $CD26^{-}$, $CD49f^{+}$ $CD26^{+}$, and $CD49f^{-}$ $CD26^{-}$ cells from human SMG were isolated via FACS-sorting and assessed for the expression of EGFR family gene members (n = 3). **(f)** Organoids from $CD49f^{+}$ $CD26^{-}$ and $CD49f^{+}$ $CD26^{+}$ cells were maintained in the GEM for 3 weeks, followed by 3 days in the DAM, and subjected to H&E staining or IF staining for duct (KRT5/KRT7), acinar (AQP5/MIST1), and myoepithelial (ACTA2/KRT14) markers. White arrows indicate $MIST1^{+}$ cells or $ACTA2^{+}$ $KRT14^{+}$ myoepithelial cell. Nuclei were stained with Hoechst 33342 (blue). Scale bars indicate 50 μm. Data are representative of at least three independent experiments, and presented as mean ± SEM, * $p < 0.05$, ** $p < 0.01$, *** $p < 0.001$. Source data are provided as a Source Data file.

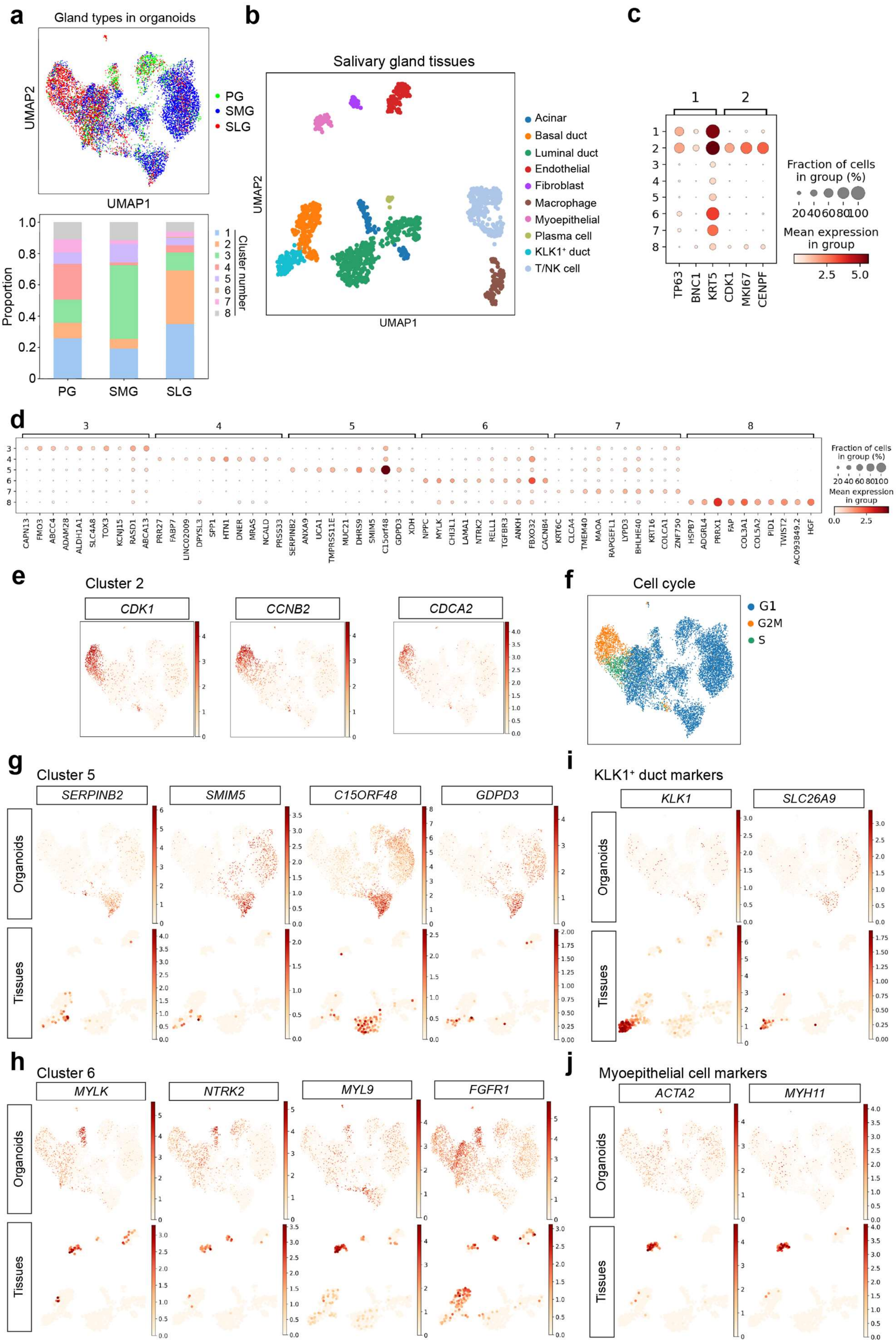

a
Gland types in organoids
UMAP2
UMAP1
PG
SMG
SLG
Proportion
Cluster number
b
Salivary gland tissues
UMAP2
UMAP1
Acinar
Basal duct
Luminal duct
Endothelial
Fibroblast
Macrophage
Myoepithelial
Plasma cell
KLK1⁺ duct
T/NK cell
c
TP63
BNC1
KRT5
CDK1
MKI67
CENPF
Fraction of cells in group (%)
Mean expression in group
d
e
Cluster 2
CDK1
CCNB2
CDCA2
f
Cell cycle
G1
G2M
S
g
Cluster 5
SERPINB2
SMIM5
C15ORF48
GDPD3
Organoids
Tissues
h
Cluster 6
MYLK
NTRK2
MYL9
FGFR1
i
KLK1⁺ duct markers
KLK1
SLC26A9
j
Myoepithelial cell markers
ACTA2
MYH11

Supplementary Figure 8. Identification of cell types in salivary gland tissues and cluster-specific gene signatures in organoids. Human PG, SMG, and SLG organoids were cultured in the GEM for 1 month and differentiated in the DAM for another 3 days. The harvested organoids were subjected to scRNA-seq. **(a)** Origins of the three major salivary gland organoids from different patients visualized as UMAP (top), with the composition of each cluster from different organoids being represented as a bar graph (bottom). **(b)** Cell clusters of human PG, SMG, and SLG tissues were visualized as UMAP (n = 1234). **(c)** Cluster-specific gene signatures of cluster 1 (basal) and cluster 2 (cycling) are displayed in dot plots. The size of the circle represents the percentage of a cell population, while its color depicts gene expression. **(d)** Cluster-specific gene signatures of clusters 3, 4, 5 (differentiated), clusters 6, 7 (transitional), and cluster 8 (mesenchymal) are displayed in dot plots. The size of the circle represents the percentage of a cell population, while its color depicts gene expression. **(e)** Genes associated with cell cycle and proliferation (*CDK1*, *CCNB2*, and *CDCA2*) were enriched in cluster 2 of salivary gland organoids, as visualized in UMAPs. **(f)** Cell cycle stages of human salivary gland organoids were visualized in UMAP. **(G)** $KLK1^{+}$ or luminal duct-specific markers (*SERPINB2, SMIM5, C15ORF48,* and *GDPD3*) were enriched in cluster 5 of salivary gland organoids (top) and tissues (bottom) visualized in UMAPs. **(h)** Expressions of cluster 6-specific genes (*MYLK*, *NTRK2*, *MYL9*, and *FGFR1*) of salivary gland organoids (top) and tissues (bottom) visualized in UMAPs. **(i)** Expression patterns of genes signature specific to the $KLK1^{+}$ duct in salivary gland tissue clusters were visualized in salivary gland organoids (top) and tissues (bottom) as UMAPs. **(j)** Expression patterns of genes signature specific to myoepithelial cell in salivary gland tissue clusters were visualized in salivary gland organoids (top) and tissues (bottom) as UMAPs.

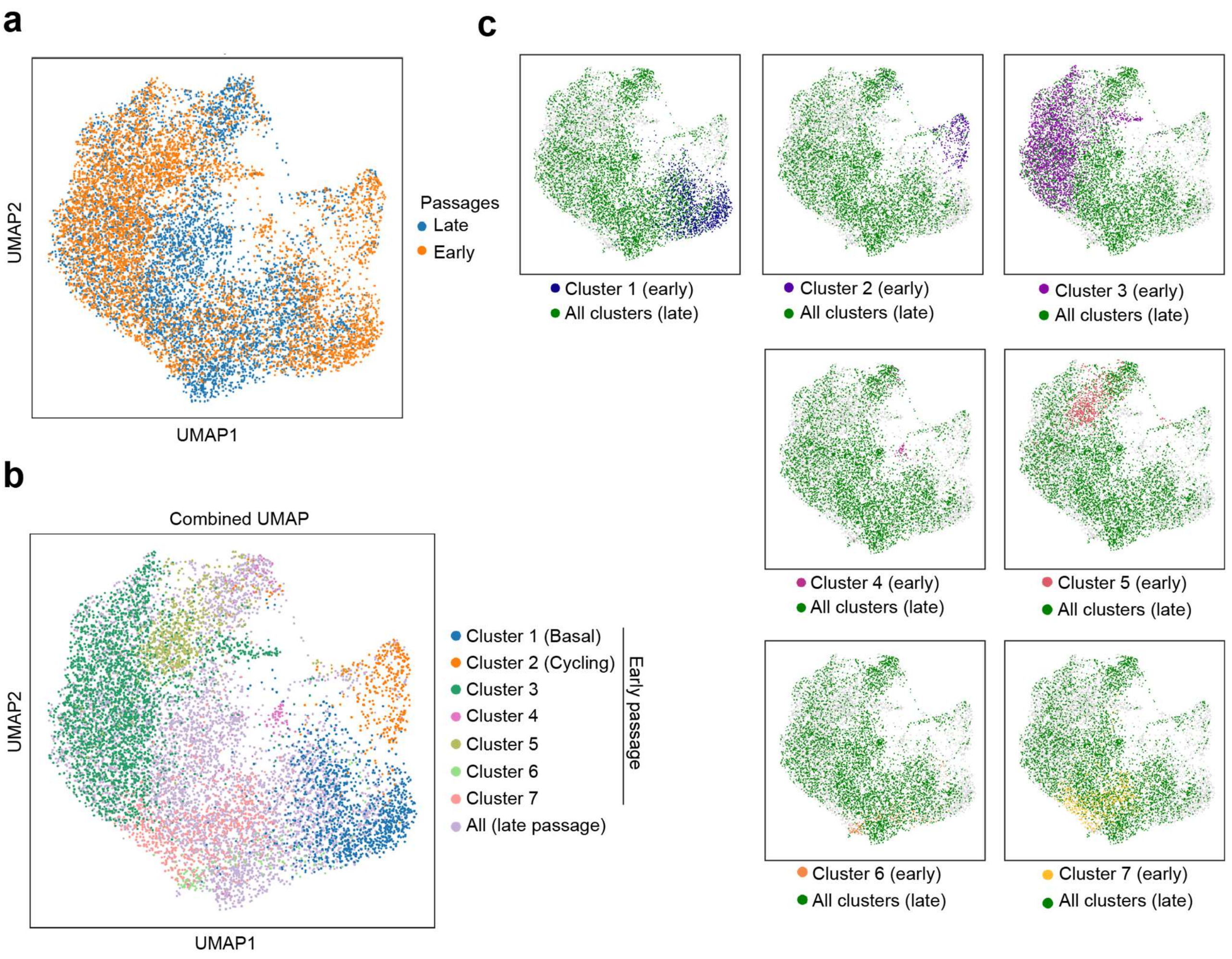

Supplementary Figure 9. Conserved clusters of human salivary gland organoids during long-term culture.

Human SMG organoids were cultured in the GEM for 1 month or 3 months, and differentiated in the DAM for another 3 days. The harvested organoids were subjected to scRNA-seq, and clusters were visualized as UMAP. **(a)** The different culture periods were illustrated with different colors (early for 1 month, orange; late for 3 months, blue). **(b)** Based on the clustering strategy used in Figure 5A, clusters from early passage were displayed with all cells from the late passage. Cluster 8 in early passage was not detected in the late passage. **(c)** Each cluster from early passage was illustrated with all cells from the late passage.

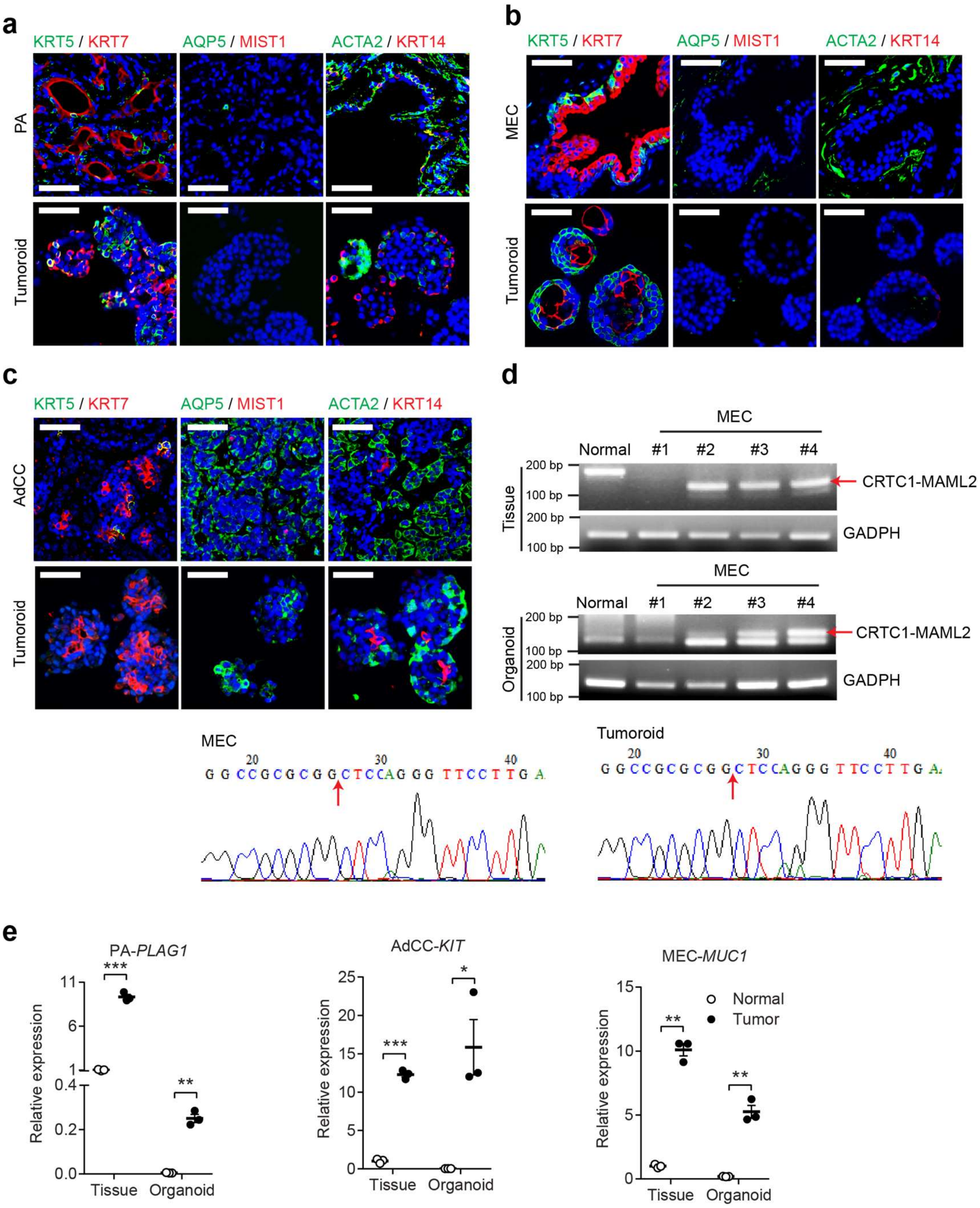

Supplementary Figure 10. Characteristics of salivary gland tumoroids.

(a-c) Pleomorphic adenoma (PA, **a**), mucoepidermoid carcinoma (MEC, **b**), or adenoid cystic carcinoma (AdCC, **c**) organoids were maintained in tumor-GEM for at least 1 month and subjected to IF staining for ductal, acinar, and myoepithelial markers (bottom). Data are displayed alongside images from matched tumor tissues (top). Nuclei were stained with Hoechst 33342. Scale bars indicate 50 μm. **(d)** MEC-specific CRTC1-MAML2 fusion gene transcripts in tumor tissues and tumoroids (n = 4) (top) validated via sequencing (bottom). Red arrows in sequencing data indicate the breakpoint between the CRTC1 and MAML2 exons. **(e)** mRNA expressions of *PLAG1* in PA (left), *KIT* in AdCC (middle), and *MUC1* in MEC (right) were determined with tissues and organoids (n = 3). Expressions at adjacent normal tissues were used as control and normalized as 1. Data are representative of at least three independent experiments, and presented as mean ± SEM, * $p < 0.05$, ** $p < 0.01$, *** $p < 0.001$. Source data are provided as a Source Data file.

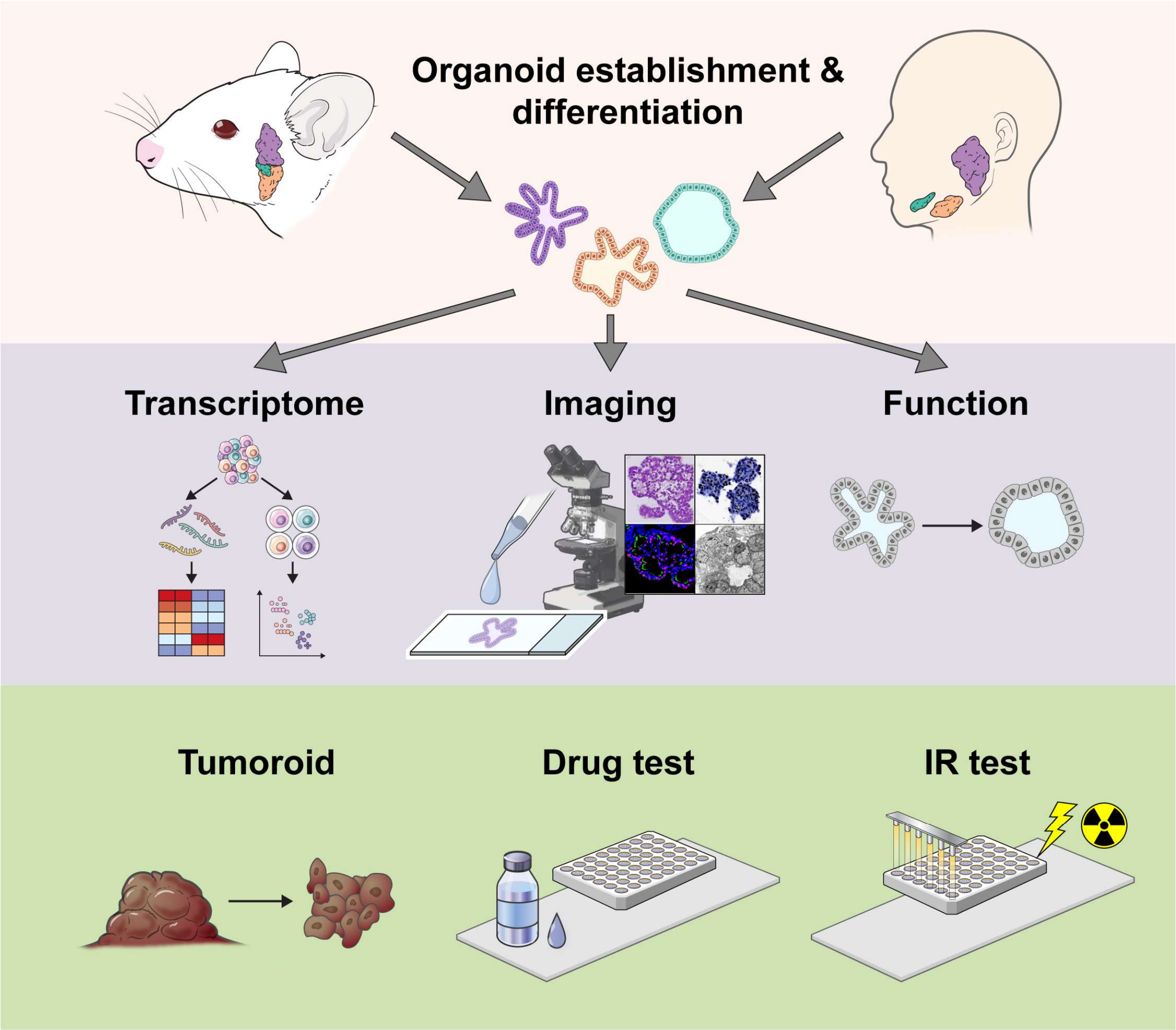

Supplementary Figure 11. Graphical summary for salivary gland organoids and their applications

Supplementary Tables

Supplementary Table 1. Formulation of media for murine salivary gland organoids

Components	Mouse GEM	Mouse DAM
HEPES	10 mM	10 mM
GlutaMAX	1×	1×
Primocin	0.2 µg/mL	0.2 µg/mL
B-27	1×	1×
N-acetyl cysteine	1.25 mM	1.25 mM
RSPO1-CM	1 %	1 %
Noggin	100 ng/mL	100 ng/mL
NRG1	5 nM	5 nM
FGF1	5 nM	5 nM
FGF7	1 nM	1 nM
A83-01	0.5 µM	0.5 µM
Y-27632	10 µM	—
DAPT	—	5 µM

GEM, growth and expansion media; DAM, differentiation-accelerating media; NRG1, neuregulin 1; FGF, fibroblast growth factor

Supplementary Table 2. Formulation of media for human salivary gland organoids and tumoroids

Components	human GEM	human DAM	tumor GEM
HEPES	10 mM	10 mM	10 mM
GlutaMAX	1×	1×	1×
Primocin	0.2 μg/mL	0.2 μg/mL	0.2 μg/mL
B-27	1×	1×	1×
N-acetyl cysteine	1.25 mM	1.25 mM	1.25 mM
RSPO1-CM	1 %	1 %	1 %
Noggin	100 ng/mL	—	100 ng/mL
NRG1	5 ng/mL	5 ng/mL	50 ng/mL
FGF2	5 ng/mL	5 ng/mL	5 ng/mL
FGF10	10 ng/mL	10 ng/mL	10 ng/mL
A83-01	5 μM	0.5 μM	0.5 μM
Nicotinamide	10 mM	—	10 mM
Prostaglandin E2	3 μM	—	3 μM
CHIR99021	1 μM	—	1 μM
Y-27632*	10 μM	—	10 μM
DAPT	—	5 μM	—

* Y-27632 was added only for the first 2-3 d after subculturing.

GEM, growth and expansion media; DAM, differentiation-accelerating media; NRG1, neuregulin 1; FGF, fibroblast growth factor

Supplementary Table 3. Gene-specific primer list

Mouse genes	Forward primers (5′-3′)	Reverse primers (5′-3′)
Wnt3a	AAC TGC ACC ACC GTC AGC AAC A	AGC GTG TCA CTG CGA AAG CTA C
Wnt4	GAG AAC TGG AGA AGT GTG GCT G	CTG TGA GAA GGC TAC GCC ATA G
Wnt5a	GGA ACG AAT CCA CGC TAA GGG T	AGC ACG TCT TGA GGC TAC AGG A
Wnt5b	GCT ACC GCT TTG CCA AGG AGT T	CAT TTG CAG GCG ACA TCA GCC A
Wnt7a	TTC GCC AAG GTC TTC GTG GAT G	TAC AGG AGC CTG ACA CAC CAT G
Wnt7b	TTC TCG TCG CTT TGT GGA TGC C	CAC CGT GAC ACT TAC ATT CCA GC
Wnt10a	GCT CCT GTT CTT CCT ACT GCT G	ATG TCA GGC ACA CTG TGT TGG C
Wnt10b	ACC ACG ACA TGG ACT TCG GAG A	CCG CTT CAG GTT TTC CGT TAC C
Ivl	ACC TGA ACC AGA ACT GCA CC	TGG CTC ACC AAG TTT CTG CT
Smgc	CTG ACA GAG GAT CAT GGA CCA AC	TCC TGA CAC CTT GGA GAG TCC A
Prol1	CAC CTA AGC CTA GCA CCT CTA	ACT TCC AAA ACA CTT CCG CAA AT
Muc19	CTG GGT CTG GAA GTA GAA GTA	TCT AAG CCA CAG AAG GAG AT
Amy2B	GAT CTG GGT GGT GAG GCA AT	TGC GGA TAA CTG TGC CAA GT
Krt5	TCC TGT TGA ACG CCG CTG AC	CGG AAG GAC ACA CTG GAC TGG
Krt7	GCT CTC GCT CCA CTG CTT AC	CGC CAG CAA GCT CTG ATT GA
Aqp5	CGC TCA GCA ACA ACA CAA CA	GAA AGA TCG GGC TGG GTT CA
Bhlha15	GCT GAC CGC CAC CAT ACT TAC	TGT GTA GAG TAG CGT TGC AGG
Krt14	GCC CAC CTT TCA TCT TCC CAA T	TTG GTG GAG GTC ACA TCT CTG G
Acta2	TGC TGA CAG AGG CAC CAC TGA A	CAG TTG TAC GTC CAG AGG CAT AG
Dcpp3	GGT CCA GAA GTT GGA AAA CAT TCC	GAG CCA TAC ACT TGA CCG TCCT
Sox2	CAG CAT GTC CTA CTC GCA GCA G	TGG AGT GGG AGG AAG AGG TAA CC
Calml3	TCC GAG TGT TCG ACA AGG ATG G	CAT TTC GTC CAC TTC CTC ATC GC
Serpine2	ACT GTC TGC CAT CAT CCC TCA C	GTA ATG CCA AGG GCT TTC AGT GG
Itga8	CCG ATT TGC TGT TCC TCG CCT T	GAC CTG AGC AAT GGC AGT GAT G
**Human genes**	**Forward primers (5′-3′)**	**Reverse primers (5′-3′)**
KRT5	GCT GCC TAC ATG AAC AAG GTG G	ATG GAG AGG ACC ACT GAG GTG T

KRT14	TGC CGA GGA ATG GTT CTT CAC C	GCA GCT CAA TCT CCA GGT TCT G
KRT7	TGT GGA TGC TGC CTA CAT GAG C	AGC ACC ACA GAT GTG TCG GAG A
AQP5	TAC GGT GTG GCA CCG CTC AAT G	AGT CAG TGG AGG CGA AGA TGC A
BHLHA15	CGG ATG CAC AAG CTA AAT AAC G	GCC GTC AGC GAT TTG ATG TAG
ACTA2	CTA TGC CTC TGG ACG CAC AAC T	CAG ATC CAG ACG CAT GAT GGC A
EGFR	AAC ACC CTG GTC TGG AAG TAC G	TCG TTG GAC AGC CTT CAA GAC C
ERBB2	GGA AGT ACA CGA TGC GGA GAC T	ACC TTC CTC AGC TCC GTC TCT T
ERBB3	CTA TGA GGC GAT ACT TGG AAC GG	GCA CAG TTC CAA AGA CAC CCG A
ERBB4	GGA GTA TGT CCA CGA GCA CAA G	CGA GTC GTC TTT CTT CCA GGT AC
PLAG1	GGT TCA CTC CTA CTC TCA CAC AG	CAG GAG AAT GAG TAG CCA TGT GC
KIT	CAC CGA AGG AGG CAC TTA CAC A	TGC CAT TCA CGA GCC TGT CGT A
MUC1	CCT ACC ATC CTA TGA GCG AGT AC	GCT GGG TTT GTG TAA GAG AGG C
