# Supplementary material for: Salivary gland organoid culture maintains distinct glandular properties of murine and human major salivary glands
Source: Nat Commun. 2022 Jun 7;13:3291. doi: 10.1038/s41467-022-30934-z (PMC9174290; doi:10.1038/s41467-022-30934-z)
Supplement: Supplementary file 3 — Description of additional Supplementary File [file 41467_2022_30934_MOESM3_ESM.pdf]

Descriptions of additional supplementary data files

Supplementary Movie 1. CChinduced swelling of human SMG organoids

Supplementary Movie 2. Isoproterenol-induced swelling of human SMG organoids

Supplementary Movie 3. VIPinduced swelling of human SMG organoids
