# Supplementary material for: Salivary gland organoid culture maintains distinct glandular properties of murine and human major salivary glands
Source: Nat Commun. 2022 Jun 7;13:3291. doi: 10.1038/s41467-022-30934-z (PMC9174290; doi:10.1038/s41467-022-30934-z)
Supplement: Supplementary file 7 — Reporting Summary [file 41467_2022_30934_MOESM7_ESM.pdf]

nature portfolio

Corresponding author(s): Jae-Yol Lim

Last updated by author(s): May 3, 2022

Reporting Summary

Nature Portfolio wishes to improve the reproducibility of the work that we publish. This form provides structure for consistency and transparency in reporting. For further information on Nature Portfolio policies, see our Editorial Policies and the Editorial Policy Checklist.

Statistics

For all statistical analyses, confirm that the following items are present in the figure legend, table legend, main text, or Methods section.

n/a	Confirmed	
☐	☒	The exact sample size (*n*) for each experimental group/condition, given as a discrete number and unit of measurement
☐	☒	A statement on whether measurements were taken from distinct samples or whether the same sample was measured repeatedly
☐	☒	The statistical test(s) used AND whether they are one- or two-sided<br>*Only common tests should be described solely by name; describe more complex techniques in the Methods section.*
☒	☐	A description of all covariates tested
☐	☒	A description of any assumptions or corrections, such as tests of normality and adjustment for multiple comparisons
☐	☒	A full description of the statistical parameters including central tendency (e.g. means) or other basic estimates (e.g. regression coefficient) AND variation (e.g. standard deviation) or associated estimates of uncertainty (e.g. confidence intervals)
☐	☒	For null hypothesis testing, the test statistic (e.g. F, t, r) with confidence intervals, effect sizes, degrees of freedom and P value noted<br>*Give P values as exact values whenever suitable.*
☒	☐	For Bayesian analysis, information on the choice of priors and Markov chain Monte Carlo settings
☒	☐	For hierarchical and complex designs, identification of the appropriate level for tests and full reporting of outcomes
☒	☐	Estimates of effect sizes (e.g. Cohen's d, Pearson's r), indicating how they were calculated

Our web collection on statistics for biologists contains articles on many of the points above.

Software and code

Policy information about availability of computer code

Data collection	FACSDiva software (9.0, BD) was used for collection of flow cytometry data.<br>Imaging data were collected via NIS-elements BR (5.20.00, Nikon) or ZEN (2.3, Carl Zeiss).
Data analysis	Statistical analysis was conducted with Graphpad Prism (Version 7).<br>FlowJo (10.7.1) was used for flow cytometry data analysis.<br>DESeq2(1.33.5) and clusterProfiler(4.1.4) were used for analysis of bulk RNAseq data.<br>CellRanger (4.0.0), Souporcell (2.0), Scanpy (1.9.1), Harmony (0.0.5), and Seurat (4.0.4) were used for analysis of single cell RNAseq data.

For manuscripts utilizing custom algorithms or software that are central to the research but not yet described in published literature, software must be made available to editors and reviewers. We strongly encourage code deposition in a community repository (e.g. GitHub). See the Nature Portfolio guidelines for submitting code & software for further information.

Data

Policy information about availability of data

All manuscripts must include a data availability statement. This statement should provide the following information, where applicable:

- Accession codes, unique identifiers, or web links for publicly available datasets
- A description of any restrictions on data availability
- For clinical datasets or third party data, please ensure that the statement adheres to our policy

The RNA sequencing data generated in this study have been deposited in the NCBI's Gene Expression Omnibus database under accession code GSE184091 (https://

www.ncbi.nlm.nih.gov/geo/query/acc.cgi?acc=GSE184091) for bulk RNA-seq or GSE184526 (https://www.ncbi.nlm.nih.gov/geo/query/acc.cgi?acc=GSE184526) for scRNA-seq. Source data are provided with this paper.

Field-specific reporting

Please select the one below that is the best fit for your research. If you are not sure, read the appropriate sections before making your selection.

- [x] Life sciences
- [ ] Behavioural & social sciences
- [ ] Ecological, evolutionary & environmental sciences

For a reference copy of the document with all sections, see nature.com/documents/nr-reporting-summary-flat.pdf

Life sciences study design

All studies must disclose on these points even when the disclosure is negative.

Sample size	No statistical methods were used to predetermine sample size. The technical and biological independent numbers were chosen according to the investigators' prior experiences and criteria accepted in other reports and scientific society in this field.
Data exclusions	In bulk RNAseq, zero-count genes in at least 1 sample were excluded for further analysis, since these genes might be expressed at very low level and did not significantly affect global profiling. In single cell RNAseq, cells with less than 1,500 measured genes were assumed as low quality and excluded. Also, cells with high percentage of mitochondrial contamination (more than 20%) were excluded for further analysis. The percentage of mitochondrial contamination was calculated with function in Seurat package and criteria was determined with outliers in plots.
Replication	All experiments were repeated at least three times (3 different experiments for organoids) showing similar results. All attempts at replication were successfull and are shown, n is described in legends.
Randomization	The experiments were not randomized as this study did not contain in vivo animal experiments or human cohorts. Samples were organized into group based on treatment and time periods. Appropriate controls were included in all experiments.
Blinding	The investigators were not blinded to allocation during experiments and outcome assessment. Blinding was not possible as the same investigator performed and analysed the data.

Reporting for specific materials, systems and methods

We require information from authors about some types of materials, experimental systems and methods used in many studies. Here, indicate whether each material, system or method listed is relevant to your study. If you are not sure if a list item applies to your research, read the appropriate section before selecting a response.

Materials & experimental systems

n/a	Involved in the study	
[ ]	[x]	Antibodies
[x]	[ ]	Eukaryotic cell lines
[x]	[ ]	Palaeontology and archaeology
[ ]	[x]	Animals and other organisms
[ ]	[x]	Human research participants
[x]	[ ]	Clinical data
[x]	[ ]	Dual use research of concern

Methods

n/a	Involved in the study	
[x]	[ ]	ChIP-seq
[ ]	[x]	Flow cytometry
[x]	[ ]	MRI-based neuroimaging

Antibodies

Antibodies used	For primary antibodies used in IHC or IF; anti-KRT5 (#905904, 1:1000; Biolegend, San Diego, CA, USA), anti-KRT7 (ab181598, 1:1000; Abcam), anti-ACTA2 (ab124964, 1:500; Abcam), anti-MIST1 (ab187978, 1:200; Abcam), anti-AQP5 (sc-514022, 1:500; Santa Cruz, Dallas, CA, USA), anti-KRT14 (ab7800, 1:200; Abcam), anti-AMY1 (sc-46657, 1:200; Santa Cruz), anti-Cleaved Caspase-3 (#9661, 1:500; Cell Signaling, Danvers, MA, USA), anti-TJP1 (#33-9100, 1:50; Invitrogen), anti-CDH1 (AF648, 1:200; R&D), anti-AMY2 (ab21156, 1:500; Abcam), anti-MUC19 (OAEB02736, 1:500; Aviva Systems Biology, San Diego, CA, USA), anti-MUC7 (orb101843, 1:400; Biorbyt, Cambridge, UK), anti-CD49f (sc-10730, 1:200; Santa Cruz), anti-CD26 (TA500733, 1:100; Origene, Rockville, MD, USA), anti-PLAG1 (H00005324-M02, 1:100; NOVUS, Centennial, CO, USA), anti-c-Kit (sc-5535, 1:100; Santa Cruz), anti-MUC1 (#14161, 1:200; Cell Signaling), and anti-p53 (#48818, 1:100; Cell Signaling).<br>For secondary antibodies used in IF; Donkey anti-Mouse IgG, Alexa Fluor™ Plus 488 (A32766, Invitrogen), Donkey anti-Rabbit IgG, Alexa Fluor™ Plus 488 (A32790, Invitrogen), Donkey anti-Mouse IgG, Alexa Fluor™ Plus 555 (A32773, Invitrogen), Donkey anti-Rabbit IgG, Alexa Fluor™ Plus 555 (A32794, Invitrogen), Donkey anti-Goat IgG, Alexa Fluor™ Plus 555 (A32816, Invitrogen), Donkey anti-Rabbit IgG, Alexa Fluor™ Plus 647 (A32795, Invitrogen), Alexa Fluor 488 AffiniPure Donkey Anti-Chicken IgY (#703545155, Jackson ImmunoResearch, West Grove, PA, USA).<br>For flow cytometry; anti-CD45-BV605 (#368524, 1:50, Biolegend), anti-CD31-FITC (#303104, 1:20, Biolegend), anti-CD49f-PE-Cy7 (#313621, 1:20, Biolegend), and anti-CD26-PE (#302705,1:20, Biolegend).
Validation	Antibodies specificity was controlled by using sections of salivary gland tissues or tumor/cancer tissue where expression of antigen have been reported. We also checked antibody application and availability in manufacturer's websites and found that antibodies were validated for the species and applications by manufacturers.

Animals and other organisms

Policy information about studies involving animals; ARRIVE guidelines recommended for reporting animal research

Laboratory animals	6 to 8 week-old female C57BL/6 mice. Mice were fed ad libitum and maintained in specific pathogen free condition at 22 +/- 2 celsius degree, 50 +/- 10% relative humidity on 12 hours of light-dark cycle (8 am - 8 pm) in facility of Yonsei University College of Medicine.
Wild animals	this study does not include wild animal usage.
Field-collected samples	this study does not include samples from field.
Ethics oversight	Institutional Animal Care and Use Committee in Yonsei University College of Medicine (approval number #2018-0071)

Note that full information on the approval of the study protocol must also be provided in the manuscript.

Human research participants

Policy information about studies involving human research participants

Population characteristics	Gender: Male (5/17), Female (12/17)<br>Age: mean 51.0, range 32 - 79<br>Tumor: Benign (6/17), Malignant (11/17)
Recruitment	Human salivary gland specimens were obtained from patients with various diseases, including benign and malignant tumors, after acquiring their informed consents. No bias on selecting patients existed.
Ethics oversight	Institutional Review Board of the Yonsei University Severance Hospital

Note that full information on the approval of the study protocol must also be provided in the manuscript.

Flow Cytometry

Plots

Confirm that:

- [x] The axis labels state the marker and fluorochrome used (e.g. CD4-FITC).
- [x] The axis scales are clearly visible. Include numbers along axes only for bottom left plot of group (a 'group' is an analysis of identical markers).
- [x] All plots are contour plots with outliers or pseudocolor plots.
- [x] A numerical value for number of cells or percentage (with statistics) is provided.

Methodology

Sample preparation	human salivary gland tissue (PG, SMG, or SLG) were digested with Collagenase type II and TrypLE Express.
Instrument	BD LSRFortessa, BD FACSAria II

Software	FlowJo for analysis, FACS Diva for data collection
Cell population abundance	we confirmed that all post-sort cells have purity more than 95% and used for experiments
Gating strategy	Singlets were gated and live cells were gated via ZombieViolet (Biolegend)-negative population. CD31-CD45 double negative population was gated as epithelial cells (non-endothelial, non-immune). Then, based on CD49f and CD26 expressions, double-positive populations were assumed as luminal, and CD49f-positive populations as basal.

☒ Tick this box to confirm that a figure exemplifying the gating strategy is provided in the Supplementary Information.
